# A tool for design of primers for microRNA-specific quantitative RT-qPCR

**DOI:** 10.1186/1471-2105-15-29

**Published:** 2014-01-28

**Authors:** Peter K Busk

**Affiliations:** 1 Department of Biotechnology, Chemistry and Environmental Engineering, Aalborg University Copenhagen, A.C. Meyers Vænge 15, Copenhagen, SV 2450, Denmark

**Keywords:** microRNA, Quantitative PCR, Primer design, Software

## Abstract

**Background:**

MicroRNAs are small but biologically important RNA molecules. Although different methods can be used for quantification of microRNAs, quantitative PCR is regarded as the reference that is used to validate other methods. Several commercial qPCR assays are available but they often come at a high price and the sequences of the primers are not disclosed. An alternative to commercial assays is to manually design primers but this work is tedious and, hence, not practical for the design of primers for a larger number of targets.

**Results:**

I have developed the software miRprimer for automatic design of primers for the method miR-specific RT-qPCR, which is one of the best performing microRNA qPCR methods available. The algorithm is based on an implementation of the previously published rules for manual design of miR-specific primers with the additional feature of evaluating the propensity of formation of secondary structures and primer dimers. Testing of the primers showed that 76 out of 79 primers (96%) worked for quantification of microRNAs by miR-specific RT-qPCR of mammalian RNA samples. This success rate corresponds to the success rate of manual primer design. Furthermore, primers designed by this method have been distributed to several labs and used successfully in published studies.

**Conclusions:**

The software miRprimer is an automatic and easy method for design of functional primers for miR-specific RT-qPCR. The application is available as stand-alone software that will work on the MS Windows platform and in a developer version written in the Ruby programming language.

## Background

MicroRNAs are non-coding RNAs that regulate gene expression and natural and disease-related cellular processes such as differentiation and cancer [1-3]. Quantification of microRNAs can be done by Illumina sequencing, DNA microarrays, Nanostrings or quantitative RT-qPCR [4]. Although all four methods are used for screening purposes and for miRNome analysis, quantitative RT-qPCR is normally the method of choice for confirming the data obtained by other methods [5]. This is due to the high sensitivity and precise and specific quantification that can be obtained in a qPCR reaction. Moreover, microRNA RT-qPCR is a popular method for development of diagnostic assays due to the high performance [6].

The design of primers for microRNA RT-qPCR is challenging as the average microRNA is only 22 nucleotides long, which is the same length as a traditional PCR primer. However, several methods have been developed to overcome this problem. All of these methods are based on elongation of the microRNA to produce a template long enough to allow the design of two primers [7]. Whereas some of the methods only use one specific primer, the stem-loop RT-PCR with a specific primer and a specific detection probe [8] and the miR-specific RT-qPCR with two specific primers [9] have the advantage that these methods use two specific oligos, which allows for high specificity and increased flexibility in primer design.

**Figure 1:**
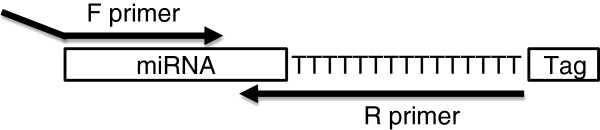
**Position of primers for miR-specific RT-qPCR.** The sequence of the forward primer (F primer) is identical to 12 – 18 nucleotides of the microRNA and may include a tag at the 5′-end. The reverse primer (R primer) is complementary to 3 – 8 nucleotides of the microRNA, followed by 15 T residues and a tail of varying length. The 15 T’s and the tail are identical to part of the RT primer sequence.

Although the microRNAs are only 22 bases long it is possible to design two, microRNA-specific primers by designing one, 12 – 18 nucleotides long forward primer and a reverse primer with 3 – 8 specific nucleotides at the 3′-end and an extension that is complementary to a universal tag, which is added to the template during cDNA synthesis (Figure 1). In the original method the primers are spiked with LNA [10] but the same specificity can be achieved with DNA primers with optimized melting temperatures [9]. Furthermore, the amplification efficiency of microRNA-specific qPCR with DNA primers is higher than with LNA-spiked primers and DNA primers are easier to design.

Hitherto, the use of miR-specific RT-qPCR for large-scale projects has been hampered by the lack of algorithms for primer design. Hence, it has been necessary to design each primer set manually. Here, I present the algorithm miRprimer for design of primers for miR-specific RT-qPCR. The algorithm generates a number of putative primers based on an interpretation of the guidelines for manual primer design [9,11] into computer language. Each primer and primer pair are assigned a score for each of the features that are relevant for performance in PCR. The output consists of a list of primer pairs ranked according to score.

Primers designed with this algorithm were tested in different experiments and have the same success rate as manually designed primers but can be made much faster.

## Implementation

Detailed guidelines for manual design of primers for miR-specific RT-qPCR have been published [9,11]. Basically, the design of a primer consists of finding the best possible 3′-end sequence for the primer and then make the primer longer towards the 5′-end until a Tm of 59°C is reached. The primer can be elongated with a tail of additional bases at the 5′-end if the microRNA template is too short to construct a primer with a Tm of 59°C.

**Figure 2:**
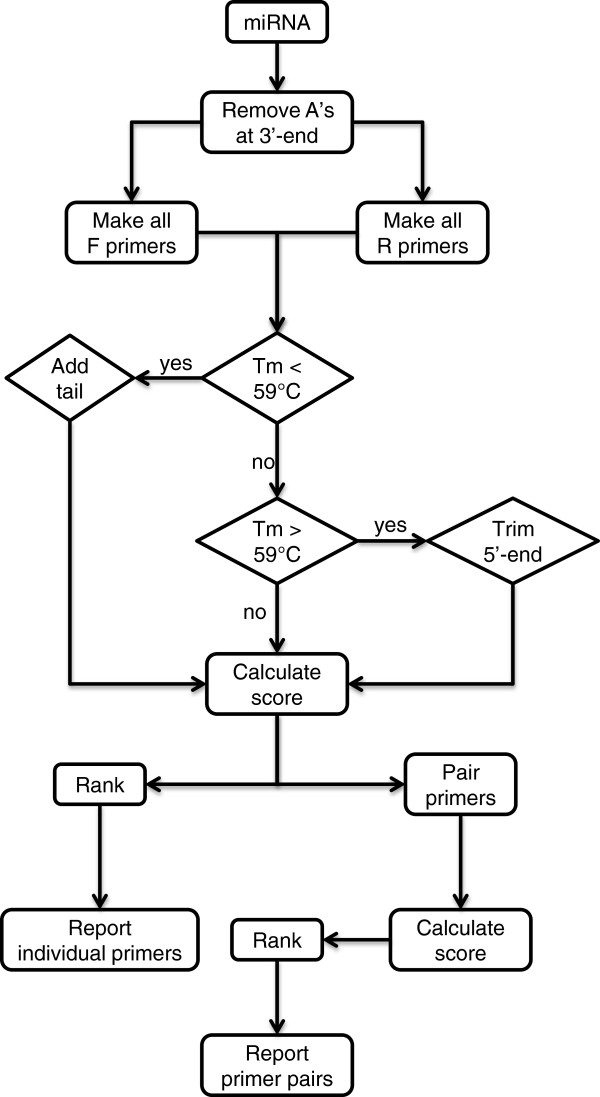
**Flow chart of how miRprimer designs primers.** F primer: forward primer; R primer: reverse primer; A: adenine residues; Tm: melting temperature.

The algorithm miRprimer was written according to the same rules but in a different order (Figure 2). Furthermore, miRprimer also takes primer secondary structures into account by calculating the propensity for the formation of primer dimers and for primer self-annealing. The first step of the algorithm is to design all putative primers with the correct Tm. Next, the primers are assigned a score according to parameters such as the sequence of the 2 – 5 nucleotides closest to the 3′-end, length of the miR-specific part of the primer and putative secondary structures (Table 1). Finally, forward and reverse primers are combined in all possible pairs and assigned a score by combining the score of each of the two primers with a score for the propensity for primer dimer formation.

**Table 1:** Parameters used in miRprimer for calculation of secondary structure score

**Parameter**	**Values**	**Score**
2 nucleotides at 3′-end	0 or 2 Ws^a^	0.7
	1 W^a^	1.0
3 nucleotides at 3′-end	0 or 3 Ws^a^	0.3
	1 or 2 Ws^a^	1.0
	0 or 5 Ws^a^	0.1
5 nucleotides at 3′-end	1 or 4 Ws^a^	0.5
	2 or 3 Ws^a^	1.0
3′-self-annealing or primer dimer	5 nucleotides	0.1
	4 nucleotides	0.2
	Less	1.0
Internal self-annealing or primer dimer	8 nucleotides	0.1
	7 nucleotides	0.3
	6 nucleotides	0.8
	Less	1.0

^a^W: A or T.

The cDNA template used for miR-specific RT-qPCR will always have 15 T’s at the 3′-end of the microRNA sequence (Figure 1) corresponding to the sequence of the primer used for reverse transcription (RT primer) [9,11]. Hence, the first step of miRprimer is to disregard any A residues at the 3′-end of the microRNA (Figure 2).

Next step is to make all putative forward primers consisting of the first 12 – 18 nucleotides from the 5′-end of the microRNA. The melting temperature (Tm) of each primer is calculated by the nearest neighbor method [12] with the NaCl concentration set to 115 mM. It was previously found that the Tm of the forward primer should be as close to 59°C as possible [10]. This can be achieved by adding a tail to the 5′-end of the sequence [8,9]. Therefore, nucleotides are added one at a time to the 5′-end of the primers that have a theoretical Tm lower than 59°C until the Tm reaches 59°C. For simplicity, the same nucleotides are added to all primers in the order G, A, C, G, C. The tail sequence was chosen to include as many G’s and C’s as possible to have the maximal effect on the Tm but without any stretches of poly(C) or poly(G) to avoid problems due to homopolymeric runs [13]. In the rare case that these five nucleotides are not enough to reach a Tm of 59°C, the same five nucleotides are added once more.

For primers with a Tm higher than 59°C nucleotides are removed one at a time from the 5′-end of the primer until the Tm is lowered to 59°C. The primer is assigned a five_prime_score of 10 if it is extended to the 5′-end of the miR and a five_prime_score of 5 if it is shorter. This score is included in the output but is not used to calculate the score of the forward primers (Table 2).

**Table 2:** Output format for forward primers

***ssc*-let-7a**	**tgaggtagtaggttgtatagtt**								
**Primers**									
**Name**	**Seq**	**Length**	**score**	**two_last_score**	**three_last_score**	**five_last_score**	**three_self_anneal**	**internal_self_anneal**	**five_prime_score**
**F_1**	gcagtgaggtagtaggttgt	16	0.64	1.0	1.0	1.0	1.0	1.0	10
**F_2**	gcagtgaggtagtaggttg	15	0.56	1.0	1.0	1.0	1.0	1.0	10
**F_3**	cgcagtgaggtagtaggt	13	0.42	1.0	1.0	1.0	1.0	1.0	10
**F_4**	cgcagtgaggtagtaggtt	14	0.34	0.7	1.0	1.0	1.0	1.0	10
**F_5**	gcagtgaggtagtaggttgta	17	0.25	0.7	1.0	0.5	1.0	1.0	10
**F_6**	cgcagtgaggtagtagg	12	0.25	0.7	1.0	1.0	1.0	1.0	10
**F_7**	gcagtgaggtagtaggttgtat	18	0.09	0.7	0.3	0.5	1.0	1.0	10
**F_8**	gcagtgaggtagtaggttgtata	19	0.02	0.7	0.3	0.5	0.2	1.0	10

The same procedure is followed for design of the reverse primers which consist of 3 – 8 nucleotides complementary to the 3′-end of the microRNA and with a tail consisting of 15 T’s corresponding to the poly(T) run of the RT primer [9]. In the case of the reverse primer, the Tm is adjusted to 59°C by adding the nucleotides corresponding to the tag of the RT primer one at a time.

The scores for the sequence of the 3′-end of the primers are based on the primer design guidelines that were published for LNA-spiked primers [10] and modified for DNA primers [9,11]. The recommendations in the guidelines were interpreted to provide a score matrix for different sequences at the 3′-end of the primers (Table 1). For example, the instruction “If possible, the three last bases at the 3′-end of the forward primer should include 1–2 A or T residues” [9] was interpreted to give a score of 1.0 to primers with 1–2 A or T residues in the three most 3′-end bases and a score of 0.3 for primers ending on other sequences.

All primers are assigned a score depending on the propensity to form secondary structures. These scores are assigned to be of similar magnitude as the 3′end scores of the primers. For example the scores of 0.2 for primers where the four nucleotides at the 3′-end are complementary to other sequences of the primer (Table 1) means that such a primer will not be designed in practice if alternatives with more optimal 3′-ends are available.

Next, the forward primers are assigned a score relating to the length of the primer without tail. This score is calculated as the square of the length of miR-specific part of the primer divided by 400. Hence, this score favors forward primers with a longer miR-specific sequence. The reverse primers are not assigned any score related to the length of the primer without tail as no significant correlation betw assay performance and primer length has been demonstrated in practice [9-11]. However, when two primer pairs have exactly the same score, the pair including the longest reverse primer is preferred.

Finally, all the scores for each parameter are multiplied to provide the score for the primer. The score should be interpreted relative to the scores of other putative primers for the same microRNA rather than as an absolute number that predicts the performance of the primer.

After designing the putative primers, miRprimer combines all forward primers with all reverse primers except for forward and reverse primers that overlap with two or more nucleotides at the 3′end. Three scores are calculated for the primer pairs that fulfill this criterion: 1. A score (Fprimer_anneal) for an overlap between the 3′-end of the forward primer to the sequence of the reverse primer. 2. A score (Rprimer_anneal) for an overlap between the 3′-end of the reverse primer to the sequence of the forward primer. 3. A score (primer_dimer) for internal overlap between the two primers.

The score for each primer pair is calculated as:

$$\begin{array}{lll} \text{pair}\;\text{score} & = & \text{forward}\;\text{primer}\;\text{score}\\ & & \times \text{reverse}\;\text{primer}\;\text{score}\times {\mathrm{Fprimer}}_{\text{anneal}}\\ & & \times {\text{Rprimer}}_{\text{anneal}}\times {\text{primer}}_{\text{dimer}} \end{array}$$

Finally, miRprimer ranks the primer pairs according to the pair score.

### Input for miRprimer

The input for miRprimer consists of a list of microRNA names and sequences in fasta format. The sequence can be uppercase or lowercase and be written as RNA using the letter “U” for uridine or as DNA using the letter “T” for thymidine. The list can be made in text processor and saved as a text file named input_miRs.txt in the same folder as miRprimer:

>*ssc*-let-7a

TGAGGTAGTAGGTTGTATAGTT

>*ssc*-miR-21

tagcttatcagactgatgttga

>*hsa*-miR-25-3p

CATTGCACTTGTCTCGGTCTGA

>*ssc*-mir30a-3p

CUUUCAGUCGGAUGUUUGCAGC

>*ssc*-miR-106a

AAAAGTGCTTACAGTGCAGGTAGC

>*gga*-miR146c-5p

UGAGAACUGAAUUCCAUGGACUG

>*ssc*-mir-148a-3p

TCAGTGCACTACAGAACTTTGT

>*hsa*-miR-223-3p

UGUCAGUUUGUCAAAUACCCCA

>*mmu*-miR-667-3p

ugacaccugccacccagcccaag

>*ssc*-miR-7134-3p

tgcggaacctgcggatacgg

The next step is to execute miRprimer. This can be done by double-clicking the icon of the program or by executing it from a dos window. This will generate the five output files result_best_primer_pairs.txt, result_all_primer_pairs.txt, result_f_primers.txt, result_r_primers.txt and result_comparison_of_pairs.txt. The files can be opened as spreadsheets in programs such as MS Excel or OpenOffice Calc.

The file result_best_primer_pairs.txt contains information on the score of the best primer pair for each microRNA, scores of the forward and reverse primers and the scores for primer dimer formation between the two primers (Table 3).

**Table 3:** Output format for best ranking primer pair

**Name**	**Sequence**	**Score**	**Fprimer_anneal**	**Rprimer_anneal**	**Primer_dimer**
***ssc*-let-7a**	tgaggtagtaggttgtatagtt	0.32	1.0	1.0	1.0
**F_1**	gcagtgaggtagtaggttgt	0.64			
**R_1**	ggtccagtttttttttttttttaactatac	0.5			

In case one wants to design two or more primer pairs or do not wish to use the recommended pair, the other four files can be used as a guide for choosing primers. The file result_all_primer_pairs.txt contains all the primer pairs for each of the microRNAs including the same information as result_best_primer_pairs.txt for each pair of primers (Additional file 1).

The score-based ranking and detailed scores for all the forward primers can be found in result_f_primers.txt (Table 2). Similar data for the reverse primers are found in result_r_primers.txt (Additional file 2).

The last file result_comparison_of_pairs.txt assigns a score for the difference between the primer pairs for each target by taking into account the difference in length of the miR-specific parts of the primers and the sequence of the last three nucleotides at the 3′-end of the primers (Additional file 2). Differences in the forward primer are weighted twice as much as differences in the reverse primer. Lower scores means that the primer pairs are more different and identical primer pairs have a score of 1.0.

## Results and discussion

Design of primers for microRNA PCR faces two challenges. One is the difficulty of accommodating two primers on a short template that can be solved by adding a tail to the microRNA [14]. This solution is used for miR-specific RT-qPCR (Figure 1). The other challenge is that the short template leaves very little degree of freedom for choosing the sequence of the primer. The polymerase elongates the primer from the 3′-end and it has been known for a long time that specific binding of the 3′-end of the primer is critical for the performance of PCR reactions [15]. Hence, a sound approach to achieve good PCR performance is to focus on designing primers with the best possible 3′-end [10]. This approach, as adapted to DNA primers [9] is the basis for microRNA primer design by the software miRprimer.

The major advantages of automated primer design compared to manual design are that automation speeds up the process and makes it easier to calculate the impact of primer secondary structures on PCR performance. The speed of primer design is especially important when designing primers for many templates, which occurs more often as the number of known microRNAs increase and transcriptomic studies become more frequent. Calculation of secondary structures makes it possible to take the effects of primer self-annealing and of primer dimer formation into consideration when choosing the primers and increases the likelihood of successful assay design.

**Figure 3:**
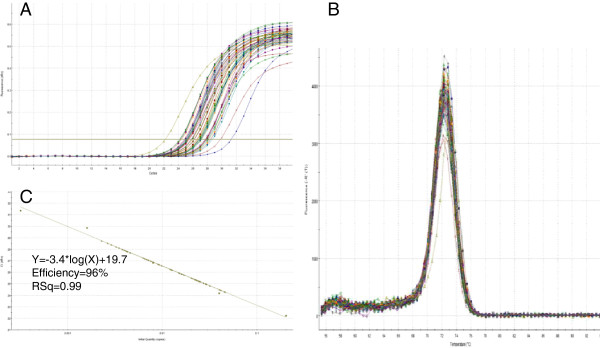
**Specific amplification of a target from a biological sample.** Detection of *ssc*-miR-15a with specific primers in cDNA made from purified pig lung total RNA (see Additional file 3). **A** Amplification curves. **B** Melting curves. **C** Extrapolation of Cq as function of the log10 of the relative number of templates was a straight line (R2 = 0.99) with a slope of -3.40 (PCR efficiency = 96%) over 4 log10 dilutions of a pool of all the samples used in the experiment.

The software miRprimer is a fast way to design primers for the method miR-specific RT-qPCR and the primers are able to perform well on targets in complex biological samples (see additional file 3) yielding typical qPCR amplification curves, melting curves with a single peak and amplification efficiencies close to 100% (Figure 3, Additional file 4). These results are similar to the performance of manually designed assays [9].

To assess the usefulness of miRprimer I collected information on the performance of primers designed by miRprimer and ranked as the best primers for the specific target microRNA (Additional file 5). The data includes results from several studies where the microRNA primers have been tested experimentally [16-20]. In total, data for 16 primer pairs and 47 single primers designed by miRprimer and predicted to be the best performing primers (highest score) combined with a manually selected primer were available. In the cases where only one of the primers was the highest ranking primer, the other primer was selected manually from the list of primers designed by miRprimer and in two cases; the forward primer was designed manually.

As shown in Table 4, 16 out of 16 assays where miRprimer predicted the primer pair to be the best pair passed the quality tests for functional assays. In total, 95 ± 7% of the forward primers and 97 ± 5% of the reverse primers suggested by miRprimer yielded functional assay (Table 4, Additional file 3). This is comparable (*P* = 0.74 for forward primers; *P* = 0.19 for reverse primers) to the 94% success rate for manually designed primers [9]. Furthermore, data were collected for 82 functional assays (Additional file 5). Out of the 164 validated primers 162 primers (99 ± 2%) were designed by miRprimer and 93 ± 5% of the assays worked with the first primer pair selected (Table 4).

**Table 4:** Success rate of primer design

**Validated assays**	**Primers designed by miRprimer**		**1**^**st **^**design successful**	
	**F primer**	**R primer**		
16	16	16	16	100%
41	41	nr^a^	39	95%
38	nr^a^	38	37	97%
84	80	81	78	93%

^a^Not relevant.

For development of several assays at a time the most rational approach is to use the primer pairs suggested by miRprimer and stored in the file result_best_primer_pairs.txt. If an assay does not work another set of primers can be chosen from the file result_all_primer_pairs.txt.

The new primer pair to be tested should not necessarily be the pair that is ranked second best by miRprimer but rather a primer pair consisting of primers that have a sequence that is as different as possible from the first primer pair that was tested. Usually, it is expected that the second highest ranking pair will also have the second highest probability of yielding a functional assay, however, it is necessary to consider why an assay fails: First, we do not have all the information about how primer sequence influence PCR performance so it is still necessary to perform some empirical testing to find the best primer pair [21]. Hence, when a given primer pair performs poorly it may indicate that one of the primers has some undesired features. Secondly, the concentrations of target and other RNA molecules that could bind the primers are seldom known for each sample. These concentrations can have different influence on the performance of each primer pair due to the different sequences of the primers. In both cases, when a primer pair fails it is advisable to design new primers that are as different as possible from the failed sequences to minimize the risk of repeating features that make the assay fail.

It is possible to access the difference between primer pairs by manual inspection. However, miRprimer generates the file result_comparison_of_pairs.txt with scores for the pairwise similarity of all primer pairs relative to each other (Additional file 6). The lower the score, the more different are the primer pairs. For example, if primer pair 1 (5′-GCAGTGAGGTAGTAGGTTGT and 5′-GGTCCAGTTTTTTTTTTTTTTTAACTATAC) do not work well for amplification of *ssc*-let-7a, it might be a good strategy to try primer pair 16 (5′-GCAGTGAGGTAGTAGGTTG and 5′-AGGTCCAGTTTTTTTTTTTTTTTAACT) where both primers are different from the initially tested primers and as pair have a similarity score of 0.16 compared to primer pair 1.

Another case where one might want to try different primer pairs than the recommended is when trying to discriminate between microRNAs with a single base difference. The closer the single base difference is to the 3′-end of the primer the larger difference between the amplification of the target and the microRNA with a single nucleotide mismatch [9]. For example, miRprimer suggests the reverse primer CCAGTTTTTTTTTTTTTTT*GGAAATCC* (microRNA-specific sequence is in italic) for amplification of *hsa*-miR-23a. However, *hsa*-miR-23a only differs from *hsa*-miR-23b in one position, which is the nucleotide four bases from the 3′-end (miRBase accession numbers: MIMAT0000078 and MIMAT0000418). Therefore, it might be better to use the reverse primer CGTCCAGTTTTTTTTTTTTTTT*GGAA* (microRNA-specific sequence is in italic) if the purpose of the study is to discriminate between *hsa*-miR-23a and *hsa*-miR-23b. However, one should always consider that the use of short primers, especially short forward primers, increase the risk of unspecific assays as a shorter part of the primer will be specific for the microRNA of interest.

**Figure 4:**
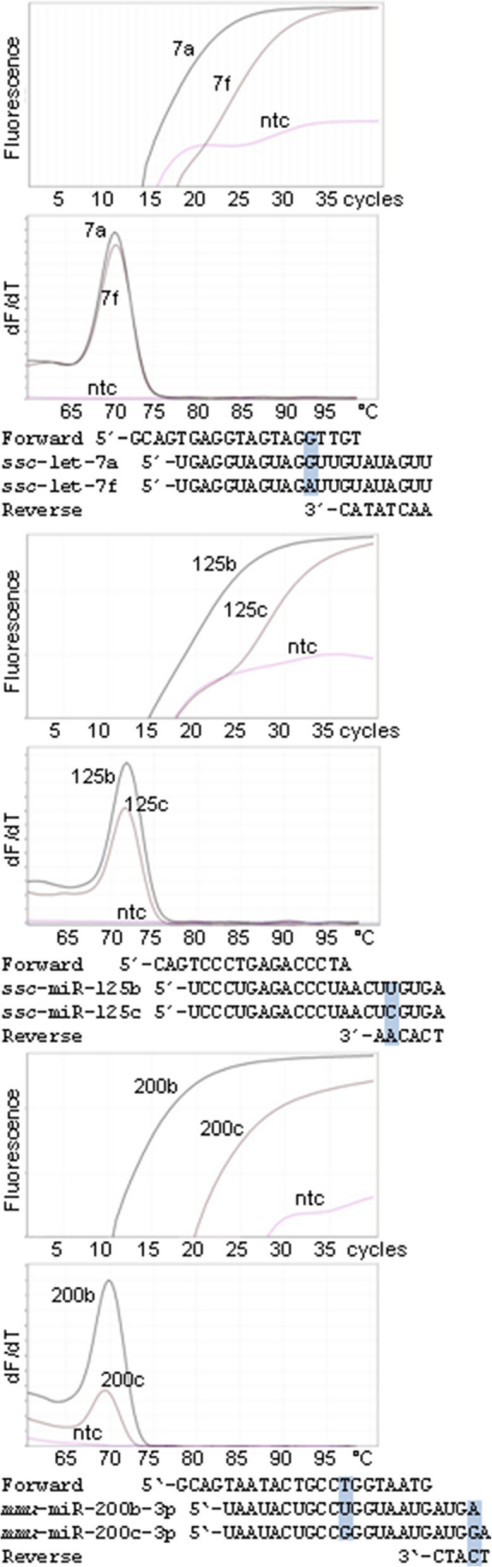
**Low detection of microRNAs that are closely related to the specific target.** Amplification plots and melting curves for amplification of specific targets (*ssc*-let-7a, *ssc*-miR-125b and *mmu*-miR-200b-3p) and closely related miRs with one base mismatch to the forward primer (*ssc*-let-7e), to the reverse primer (*ssc*-miR-125c) and to each of the primers (*mmu*-miR-200c-3p). The position of the mismatch is indicated with a box on the alignment of primers and microRNAs. Only the microRNA-specific bases of the reverse primers are shown in the alignment. The curves labeled “ntc” are non-template controls. The experiment was performed as previously described using the same primers for *ssc*-let-7a and *ssc*-miR-125b [9]. QPCR of *mmu*-miR-200c-3p was done with the Brilliant III Ultra-Fast QPCR Master Mix (Agilent, USA). The sequence of the primers can be found in Additional file 5 (*ssc*-miR-125b is identical to *mmu*-miR-125b).

Hence, short primers have higher propensity to bind to other off-target sequences than the microRNA with the single base difference. At present there is not enough information about factors affecting primer binding and practical RNA concentrations to calculate an optimal balance between designing primers that have high overall specificity but do not bind to microRNAs that are closely related to the target. Therefore, primers have to be tested experimentally. Fortunately, most primer pairs designed by miRprimer discriminate well between closely related microRNAs (Figure 4), no matter if the single nucleotide difference is located in the sequence binding to the forward primer (*ssc-*let-7a/f), the reverse primer (*ssc-*miR-125b/c) or both (*mmu-*miR-200b/c). The amplification of the off-target microRNA was 1% (*ssc-*let-7f, [9]), 0.7% (*ssc-*miR-125c, [9]) and 0.1% (*mmu-*miR-200c) respectively, of the amplification of the correct target. Thus, in most cases the need to avoid amplification of background RNA and to discriminate between closely related microRNAs is met by a single primer pair. This may be due to that the relatively low primer concentrations and low Tm of the primers compared to the annealing temperature used in the PCR amplification make the assays sensitive to single nucleotide mismatches [9].

To implement the published primer design rules in a computer algorithm it was necessary to assign quantitative scores to the rules for designing the 3′-end of the primer. One possibility was to assign scores based on previously published statistical analysis of the effect of 3′-end sequence on primer performance [21,22]. However, as noted by Onodera [21] classical design rules [15] create a strong bias on the primers that are reported and, hence, on the 3′-ends that seem to work. The only way to overcome this problem is to systematically test all possible 3′ends on the corresponding templates. However, such a study is not feasible in practice.

Therefore, the primer design rules for miR-specific RT-qPCR [9] were translated to primer scores in miRprimer according to classical rules for primer design [15] and to my own experience from primer design in general and specifically as inventor of the miR-specific RT-qPCR method [9-11]. One important point when designing primes for microRNAs is that the primers that are compared have highly similar sequence. Hence, if a microRNAs has three C’s in a row, it is not necessary to compare the theoretical performance of the three C’s to the theoretical performance of other runs of three bases in other primers as one does when it is possible to place the primers in several different regions of the template. In the case of microRNAs it is more relevant to compare the score of having the three C’s at the 3′-end to the score of not having three C’s at the 3′-end.

The scores for primer secondary structures have been assigned by the same principles as the score for the 3′-end. For example, a primer dimer involving 7 bases has a lower score than a primer dimer involving only 6 bases. The score could also be calculated by using thermodynamic parameters [23] but it is not clear whether this would improve the design in the special case of designing primers for microRNAs.

In the present study it was the aim to assign scores to different sequence features in a simple, systematic and efficient way to achieve a fast-performing and highly transparent algorithm. The present version of miRprimer has a success rate of primer design comparable to manually designed primers. However, the developer version of miRprimer allows users to change the scores assigned to different features of the primers to try to increase the success rate.

The four different output files containing a wealth of information for different purposes makes miRprimer better suited as a software tool for distribution than as a web-based interface.

## Conclusion

The software miRprimer is an easy to use tool that designs primers for PCR amplification of microRNAs with high success rate. The primers are designed to work for the conditions of the protocol miR-specific RT-qPCR [9,11] and have been tested with success in several laboratories [16,17,19,20]. Two versions of miRprimer are available: A user-friendly version (miRprimer.exe) and a developer version (miRprimer.rb) that can be easily altered to optimize the algorithm for special purposes or to modify parameters or incorporate new features.

MiR-specific RT-qPCR is an easy, specific and efficient method for qPCR of microRNAs that keeps costs to a minimum [24]. The availability of automated primer design makes this method an even more attractive option for quantification of microRNA expression.

## Availability and requirements

**Project name:** miRprimer

**Project home page:**https://sourceforge.net/projects/mirprimer/

**Operating systems:** Windows XP or higher

**Programming language:** Ruby 1.9.3

**Other requirements:** None

**License:** Apache License V2.0.

**Any restrictions to use by non-academics:** Commercial use may be restricted by third party rights.

## Competing interests

PKB is inventor of a patent on miR-specific RT-qPCR filed by Exiqon A/S. All commercial rights to method described in the patent were assigned to Exiqon A/S. Furthermore, PKB was an employee of Exiqon A/S until December, 2008 but has not had any economic interest in the company since 2009.

## Authors’ contributions

PKB set up the criteria for primer design, implemented the algorithm as a Ruby script, performed the mismatch qPCR experiments, all of the qPCR data analysis and wrote the manuscript.

## Supplementary Material

Additional file 1 **Output format for all primer pairs.** This file includes an example of the output format for all primers designed by miRprimer for a single target (*ssc*-let-7a). Click here for file

Additional file 2 **Output format for reverse primers.** This file includes an example of the output format for all reverse primers designed by miRprimer for a single target (*ssc*-let-7a). Click here for file

Additional file 3 **Description of experimental methods and data analysis.** This file includes an explanation of the experimental methods used for amplification of targets from biological samples and of the statistical analysis. Click here for file

Additional file 4 **Amplification of *ssc*-miR-15b and *ssc*-miR-200b from biological samples.** This file includes two examples of amplification of targets from biological samples. Click here for file

Additional file 5 **82 validated assays.** This file includes primer sequences and information about design for 82 validated miR-specific RT-qPCR assays. Click here for file

Additional file 6 **Output format for primer pair comparison.** This file includes an example of the output format for the comparison of the primer pairs designed by miRprimer for a single target (*ssc*-let-7a). Click here for file
