# Supplementary material for: A tool for design of primers for microRNA-specific quantitative RT-qPCR
Source: BMC Bioinformatics. 2014 Jan 28;15:29. doi: 10.1186/1471-2105-15-29 (PMC3922658; doi:10.1186/1471-2105-15-29)
Supplement: Additional file 3 — Description of experimental methods and data analysis. This file includes an explanation of the experimental methods used for amplification of targets from biological samples and of the statistical analysis. [file 1471-2105-15-29-S3.pdf]

Additional file 3 Experimental methods and statistical analysis.

RNA isolation, cDNA synthesis and qPCR

For the experiment on *ssc*-miR-15a and *ssc*-miR-15b RNA isolation, cDNA synthesis and qPCR were performed as described [18]. Briefly RNA was isolated from porcine lung tissue using miRNeasy Mini Kit (Qiagen, Germany) with DNAseI treatment according to manufacturer's recommendations.

Reverse transcription of 1 µg total RNA was performed according to Balcells *et al.* [9]. Briefly, 100 ng of total RNA were poly(A) tailed and reverse transcribed in a single tube reaction: 1 µl of 10x poly(A) polymerase buffer, 0.1 mM of ATP, 1 µM of RT-primer (5'-CAGGTCCAGTTTTTTTTTTTTTTTVN, V = A, C and G; N =A, C, G and T), 0.1 mM of dNTPs, 100 units of MuLV reverse transcriptase (New England Biolabs, USA) and 1 unit of poly(A) polymerase (New England Biolabs, USA) were mixed in a final volume of 10 µl and incubated at 42 ºC during 1 hour followed by 5 minutes at 95 ºC. The cDNA was diluted before the qPCR reaction.

QPCR analysis were performed on a MX3000P machine (Stratagene, USA) in 10 µl total volume with 1 µl of diluted cDNA, 5 µl of 2x QuantiFast SYBR Green PCR master mix (Qiagen, Germany), 250nM of each miR specific primer. QPCR primers for *ssc*-miR-15a were 5'-gcagtagcagcacataatg and 5'-ggtccagtttttttttttttttacaa described in Skovgaard *et al*. [18] and for *ssc*-miR-15b were: 5'-gcagtagcagcacatca 3' and 5'-ggtccagtttttttttttttttgtaa. Standard curves with 5-fold dilutions (made with a pool of equal amounts of cDNA stocks from all included samples) were made for both assays to calculate the PCR efficiency. Cycling conditions were 95 ºC for 5 min followed by 40 cycles of 95 ºC for 10 sec and 60 ºC 30 sec. A melting curve analysis (60 ºC to 99 ºC) was performed after the last cycle.

For the experiment on *ssc*-miR-200b, RNA isolation, cDNA synthesis, qPCR and qPCR primers gcagtaatactgcctggtaatg and ggtccagttttttttttttttcatc are described in [9].

All RT-qPCR experiments are MIQE guidelines compliant.

Statistical analysis

The fraction of functional primers (see for example Table 4) is given with as a number with a margin (for example 93 ± 5 % of the assays worked with the first primer pair selected). The number was calculated as the success rate: number of functional assays divided by the total number of assays. Next, I found the theoretical numbers of functional and failed assays that would give a P-value > 0.05 when compared to the observed numbers of functional and failed assays in a X^2-test. Finally, margins were calculated as: observed success rate – theoretical success rate.

Comparison of primer success rate to the success rate for manually designed primers [9] was done by X^2-test.
