# Supplementary material for: A tool for design of primers for microRNA-specific quantitative RT-qPCR
Source: BMC Bioinformatics. 2014 Jan 28;15:29. doi: 10.1186/1471-2105-15-29 (PMC3922658; doi:10.1186/1471-2105-15-29)
Supplement: Additional file 4 — Amplification of ssc -miR-15b and ssc -miR-200b from biological samples. This file includes two examples of amplification of targets from biological samples. [file 1471-2105-15-29-S4.pdf]

Additional file 4: Amplification of *ssc*-miR-15b and *ssc*-miR-200b from biological samples.

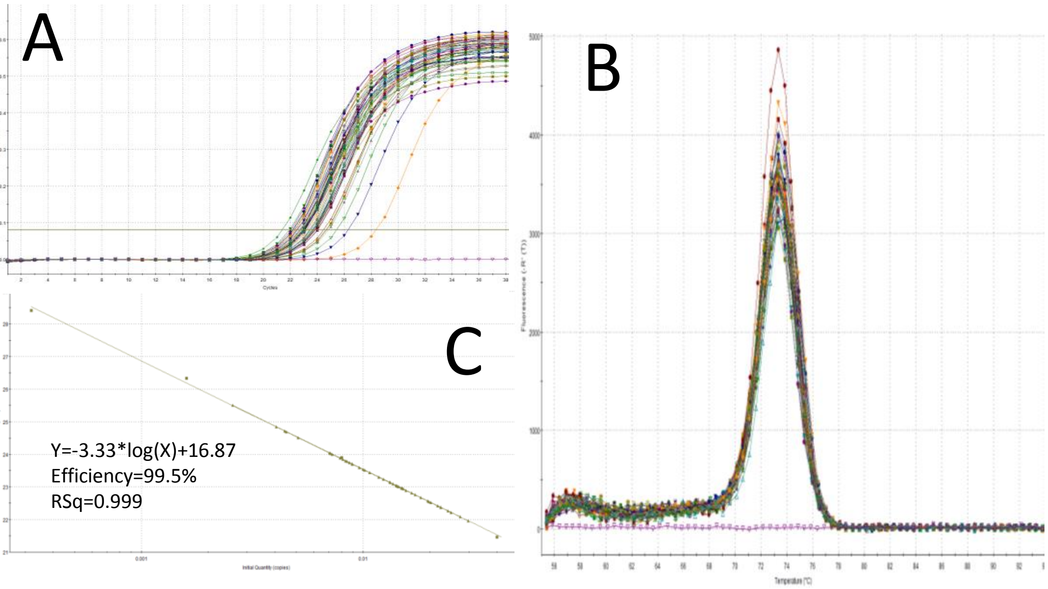

Detection of *ssc*-miR-15b with specific primers in cDNA made from purified pig lung total RNA.

A Amplification curves. B Melting curves. C Extrapolation of Cq as function of the log10 of the relative number of templates was a straight line (R2 = 0.999) with a slope of -3.33 (PCR efficiency = 99.5 %) over 4 log10 dilutions of a pool of all the samples used in the experiment. See additional file 3 for experimental methods.

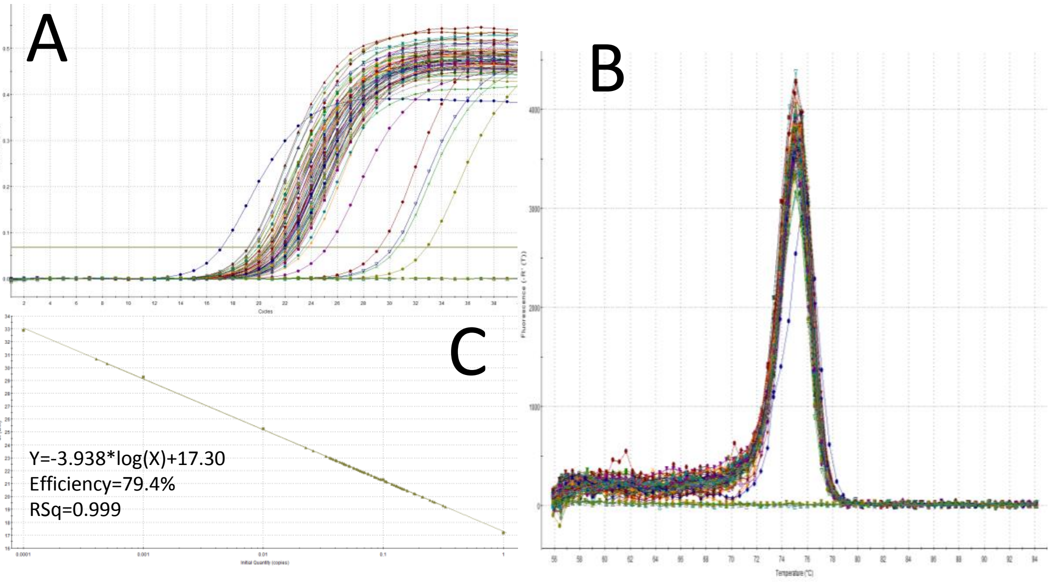

Detection of *ssc*-miR-200b with specific primers in cDNA made from purified pig uterus total RNA.

A Amplification curves. B Melting curves. C Extrapolation of Cq as function of the log10 of the relative number of templates was a straight line (R2 = 0.999) with a slope of -3.938 (PCR efficiency = 79.4 %) over 4 log10 dilutions of a pool of all the samples used in the experiment. See additional file 3 for experimental methods.
